# Three years of growth hormone treatment in young adults with Prader-Willi syndrome: sustained positive effects on body composition

**DOI:** 10.1186/s13023-020-01440-6

**Published:** 2020-06-24

**Authors:** Layla Damen, Stephany H. Donze, Renske J. Kuppens, Nienke E. Bakker, Laura C. G. de Graaff, Janielle A. E. M. van der Velden, Anita C. S. Hokken-Koelega

**Affiliations:** 1 Dutch Growth Research Foundation, Rotterdam, The Netherlands; 2 Department of Pediatrics, Subdivision of Endocrinology, Erasmus University Medical Center-Sophia Children’s Hospital, Rotterdam, the Netherlands; 3 Internal Medicine, Division of Endocrinology, Erasmus University Medical Center, Rotterdam, the Netherlands; 4 Department of Pediatrics, Subdivision of Endocrinology, Radboud University Medical Center-Amalia Children’s Hospital, Nijmegen, The Netherlands

**Keywords:** Prader Willi syndrome, Adults, Body composition, Growth hormone

## Abstract

**Background:**

In children with Prader-Willi syndrome (PWS), the benefits of growth hormone treatment are well established. Several one-year studies have shown that growth hormone is also beneficial for adults with PWS, improving body composition. However, little is known about the longer-term effects.

This study investigated the effects on body composition in adult patients with PWS during 3 years of growth hormone therapy in a dose of 0.33 mg/m^2^/day.

**Methods:**

Open-label, prospective study in 43 young adults with PWS with a median (IQR) age of 19.0 (17.5 to 20.7) years. Fat mass percentage SDS and lean body mass SDS were measured annually by DXA.

**Results:**

Estimated mean (95% CI) fat mass percentage SDS decreased during the three-year study from 2.1 (1.9 to 2.3) SDS at start to 1.9 (1.8 to 2.1) SDS, *p* = 0.012, while lean body mass SDS remained stable at − 2.1 (− 2.4 to − 1.8) SDS at start to − 1.9 (− 2.3 to − 1.6) after 3 years, *p* = 0.15. Fasting glucose and insulin remained similar during the three-year study, glucose being 4.6 (4.4 to 4.8) mmol/l at start and 4.6 (4.5 to 4.7) mmol/l after 3 years of growth hormone, *p* = 0.93 and insulin being 59.5 (42.2 to 81.5) pmol/l and 55.0 (42.4 to 69.2) pmol/l, resp., *p* = 0.54. There were no growth hormone-related adverse events during the study.

**Conclusions:**

Three years of growth hormone treatment in young adults with PWS maintains the positive effects on body composition attained during childhood. Thus, adults with PWS benefit from longer-term growth hormone treatment.

**Trial registration:**

EudraCT, EudraCT number 2011-001313-14. Registered 17 October 2012.

## Background

Prader-Willi syndrome (PWS) is a rare genetic disorder caused by the lack of expression of the imprinted genes on the Prader-Willi region of the paternally inherited chromosome 15. This is mostly caused by a paternal deletion or maternal uniparental disomy (mUPD) and in some cases by an imprinting center defect (ICD) or paternal chromosomal translocation [1, 2]. PWS is characterized by short stature, muscular hypotonia, developmental delay, behavioral problems and hyperphagia resulting in severe obesity when uncontrolled [2–4]. Body composition is abnormal with a high body fat percentage and a low lean body mass (LBM), even if there is no obesity [4–7]. Many of the symptoms in PWS may be explained by hypothalamic dysfunction.

In children with PWS, the benefits of growth hormone (GH) treatment are well established [5]. GH improves body composition, psychomotor development, cognition, adaptive functioning and linear growth [5, 8–11], without adverse effects on glucose parameters, lipid profile and blood pressure [5, 12]. As a result, GH treatment has substantially changed the phenotype of children with PWS [5, 13].

Currently, when young adults with PWS without adult growth hormone deficiency (GHD) have attained adult height (AH), they have to discontinue GH treatment because there is no reimbursement of GH for adults with PWS. However, GH studies in adults with PWS, have shown positive effects on body composition [14–19]. Our previous randomized, double-blind, placebo-controlled cross-over study (Transition Study) in 27 young adults, who were treated with GH during childhood and had attained adult height, showed deterioration of body composition during 1 year of placebo, while GH maintained the improved fat mass (FM) and LBM [14]. Other GH studies have investigated GH treatment for a maximum duration of 1 year [15–19]. However, these studies included patients not treated during childhood or in which treatment during childhood was not reported [15–17], consisted of small populations [15, 17, 18] or included only patients with adult GHD [19]. Only one study reported the effects of GH during 2 years in adults and showed positive effects on FM and LBM [20], but, GH treatment during childhood was not reported.

We report the effects of 3 years of continuous GH treatment with a stable GH dose on body composition in adults with PWS, who were treated with GH during childhood. Given the beneficial persistent effects of long-term GH on body composition during childhood, we hypothesized that GH treatment in young adults with PWS would have positive effects on body composition, also on the longer-term.

## Methods

### Patients

For the current study, we included 43 young adults participating in the Dutch Young Adult PWS (YAP) study, coordinated by the Dutch Growth Research Foundation. Inclusion criteria were (1) genetically confirmed diagnosis of PWS by a positive methylation test, (2) GH treatment for at least 5 years during childhood, (3) at least 3 years of continuous GH treatment after attainment of adult height (AH), which was defined as a height velocity less than 0.5 cm per 6 months and a complete epiphyseal fusion. Exclusion criteria were (1) medication to reduce weight (fat), (2) non-cooperative behaviour or (3) obstructive sleep apnea syndrome.

At attainment of adult height, GH treatment had to be discontinued as there is no approval for GH treatment for adults with PWS. The YAP Study was started in 2011 to evaluate the longer-term effects and safety of GH treatment in young adults with PWS who were treated with GH during childhood. Patients were included either after participation in the Dutch PWS Cohort Study in children [5, 21] or the Transition Study [14].

For patients who participated in the Dutch PWS Cohort Study (the *‘continuation group’*), the GH dose was lowered after AH attainment from 1 mg/m^2^/day to 0.33 mg/m^2^/day. Patients who participated in the Transition Study (the *‘restart group’*), had been without GH treatment for a median duration of 1 year before restart of GH in a dose of 0.33 mg/m^2^/day. The purpose of present study was to investigate the effects of 3 years of GH with a stable dose. Therefore, we did not analyse the first year after GH dose lowering of patients who participated in the Cohort Study in the 3-year analyses. For the patients who participated in the Transition study, the first year after GH restart was not analysed either, for the sake of dose stability. We chose to exclude this first year in both groups to eliminate the influence of GH dose lowering or GH restart. However, this year was used for subanalyses of the effects of continuation of GH in a lower dose in the patients in the continuation group and the effects of restart of GH in the patients in the restart group.

In girls, hypogonadism was defined as serum oestradiol levels < 100 pmol/L and/or Tanner stage 3 or less from the age of 14 years, and/or no menarche from the age of 16 years. In boys, hypogonadism was defined as morning serum testosterone levels < 5 nmol/L and/or Tanner stage 3 or less from the age of 16 years, and/or serum testosterone levels < 10 nmol/L from the age of 18 years.

The study was approved by the Medical Ethics Committee of the Erasmus University Medical Center, Rotterdam, the Netherlands. Written informed consent was obtained from participants and their legal representatives.

### Design

Open-label, prospective study investigating the longer-term effects of 3 years of GH on body composition. All participants were investigated at the Dutch PWS Reference Center in Rotterdam. Patients were treated with GH in a dose of 0.33 mg/m^2^/day (~ 0.012 mg/kg/day). The dose was adjusted based on calculated body surface area and serum IGF-I levels between 1 and 2 SDS. Patients were examined every 6 months by the PWS-team of the Dutch Growth Research Foundation in collaboration with a multidisciplinary team.

### Anthropometry

Standing height was measured in centimetres with a calibrated Harpenden stadiometer. Body weight was measured in kilograms on an electric calibrated scale (Servo Balance KA-20-150S; Servo Berkel Prior, Katwijk, The Netherlands) and body mass index (BMI) was calculated. Height, weight and BMI were expressed as standard deviations scores (SDS), adjusted for age and sex according to Dutch reference values [22, 23]; BMI also according to PWS reference values [24], using GrowthAnalyser Version 4.0 (available at www.growthanalyser.org). Systolic and diastolic blood pressure were measured using an appropriately sized cuff while patients were in sitting position.

### Body composition

FM and LBM were measured by DXA (Lunar Prodigy; GE Healthcare) in Erasmus MC, with daily quality assurance. The intra-assay coefficients of variation were 0.41–0.88% for fat tissue and 1.57–4.49% for LBM [25]. LBM was calculated as fat-free mass minus bone mineral content. FM was also expressed as percentage of total body weight (FM%). FM% SDS and LBM SDS were calculated according to age- and sex-matched Dutch reference values [26].

### Assays

Blood samples were collected after an overnight fast and measured in the Biochemical and Endocrine laboratories of the Erasmus Medical Center, Rotterdam. Fasting glucose and insulin were immediately assayed. Insulin levels were assessed using the Immulite 2000 assay (Siemens Healthcare Diagnostics). Interassay CV was 4.4%. From 2011 to 2013, serum IGF-I and IGFBP-3 levels were assessed using the Immulite 2000 (Siemens Health-care Diagnostics, Deerfield, IL), with interassay CVs of 6.5 and 8%, respectively. After 2013, IGF-I and IGFBP-3 were measured using the IDS-iSYS (Immunodiagnostic Systems), with an interassay CV of < 6.0% and < 5.1, resp. and intra-assay CV of < 2.1 and < 4.3%, resp. for IGF-I and intra-assay CV < 5.1% for IGFBP-3. Levels of IGF-I and IGFBP-3 were expressed as SDS, adjusting for age and gender [27, 28].

### Statistics

Statistical analyses were performed with SPSS version 24.0 (SPSS Inc., Chicago, IL). Variables were expressed as median (interquartile range [IQR]). Changes over time were calculated using linear mixed model analysis with the outcomes measured at each time point as dependent variable with an unstructured covariance matrix. Effects are presented as estimated marginal mean (standard error of the mean, SEM or 95% CI). Differences were considered significant if the *p*-value was < 0.05.

## Results

### Characteristics at start of the 3-year study

Forty-three young adults with PWS (18 males, 25 females) were included in the current study (Table 1). Median (IQR) age at start study was 19.5 (18.7 to 20.7) years for males and 18.4 (16.9 to 20.8) years for females. Adult height (AH) was − 1.0 (− 1.7 to − 0.3) SDS and BMI 24.5 (21.9 to 27.7) kg/m^2^. Clinical characteristics at start of the 3-year study are shown in Table 1. Eighteen (41.9%) patients had a deletion, 20 (46.5%) an mUPD, four (9.3%) an ICD and one (2.3%) a translocation. Thirty-three patients were on sex steroid replacement therapy (SSRT) during the study and ten were not. Not prescribing SSRT in these patients was a mutual decision between physician and caregivers based on patient characteristics at that moment (e.g. behavioural or weight problems). Seven patients were on thyroid medication.

**Table 1:** Clinical characteristics at start of 3-year study

	Total group
Number (females)	43 (25)
Genetic subtype	
Deletion / mUPD / ICD / translocation	18 / 20 / 4 /1
Age at start of childhood GH treatment (yrs)	7.6 (5.2 to 10.1)
Age at inclusion (yrs)	
- Males	19.5 (18.7 to 20.7)
- Females	18.4 (16.9 to 20.8)
Adult height (SDS)	−1.0 (− 1.7 to − 0.3)
BMI (kg/m^2^)	24.5 (21.9 to 27.7)
BMI for age (SDS)	0.9 (0.0 to 1.8)
BMI for PWS (SDS)	−1.4 (−2.0 to −0.7)
Fat mass percentage (SDS)^a^	2.2 (1.7 to 2.5)
Fat mass percentage (%)	40.9 (34.4 to 45.6)
Lean body mass (SDS)^a^	−2.3 (− 2.8 to −1.4)
GH-dose (mg/m^2^/day)	0.33 (0.33 to 0.59)
GH-dose (mg/kg/day)	0.012 (0.012 to 0.021)
IGF-I SDS^a^	1.5 (0.6 to 2.0)

Data expressed as median (IQR)

*mUPD* Maternal uniparental disomy, *ICD* Imprinting center defect, *GH* Growth hormone

^a^FM% SDS, LBM SDS and IGF-I SDS were calculated according to age- and sex-matched Dutch references [25]

### Changes during 3 years of GH treatment

**Fig. 1:**
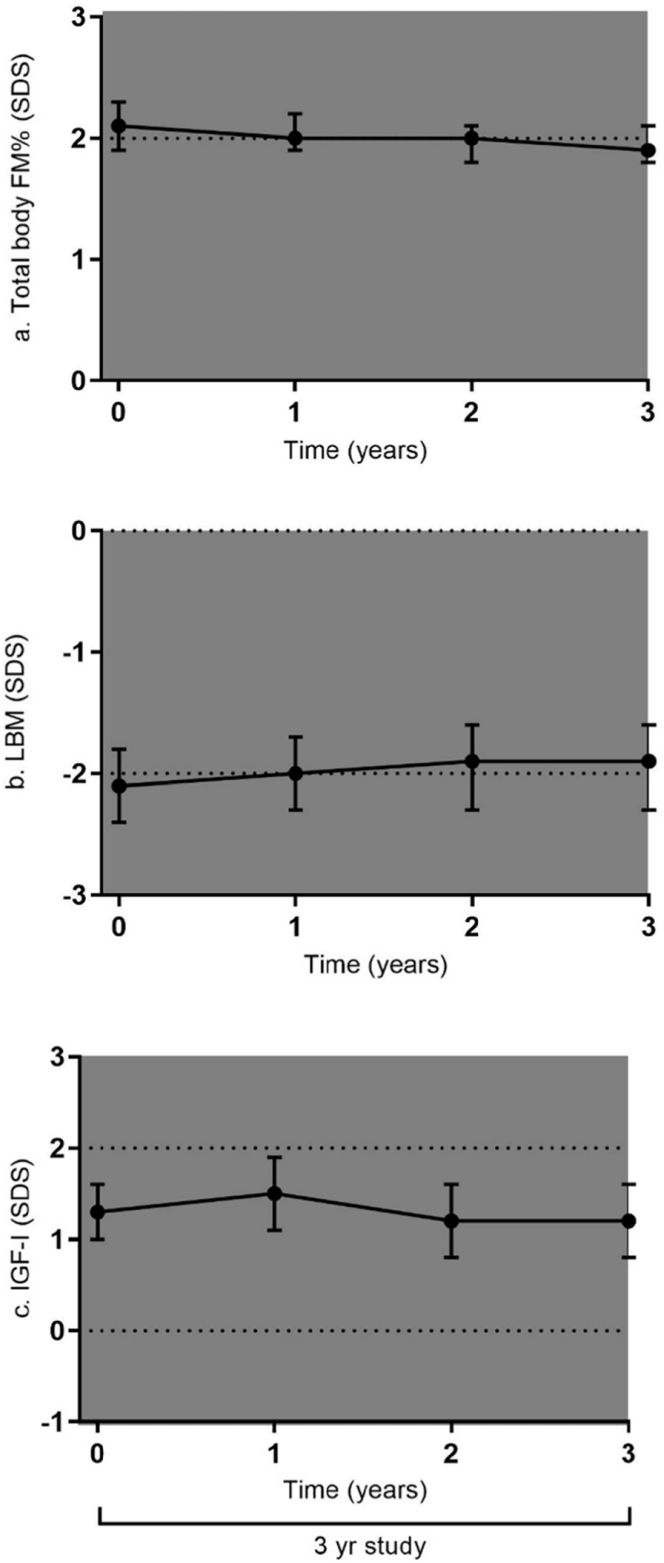
**a**-**c**. Changes in body composition in 43 young adults with PWS during 3 years of GH treatment. Presented as Estimated Marginal Means with 95% CI

Table 2 and Fig. 1a-c show the changes in body composition and IGF-I SDS during 3 years of GH treatment in the 43 young adults. Median (IQR) GH dose during the 3-year study was 0.38 (0.33 to 0.45) mg/m^2^/day. Estimated mean (95% CI) total body FM% SDS decreased during 3 years of GH treatment from 2.1 (1.9 to 2.3) SDS at start to 1.9 (1.8 to 2.1) SDS after 3 years of GH treatment, *p* = 0.012. Total body LBM SDS remained stable during 3 years of GH treatment, being − 2.1 (− 2.4 to − 1.8) SDS at start and − 1.9 (− 2.3 to − 1.6) SDS after 3 years, *p* = 0.15. During the 3 year study, IGF-I SDS remained stable, being 1.3 (1.0 to 1.6) SDS at start and 1.2 (0.8 to 1.6) SDS after 3 years. BMI SDS and BMI PWS SDS did not change during the study.

**Table 2:** Body composition during the 3-year study

	At start	After 1 year	After 2 years	After 3 years	*P*-value*
*Total group*					
Total body FM% (SDS)	2.1 (1.9 to 2.3)	2.0 (1.9 to 2.2)	2.0 (1.8 to 2.1)	1.9 (1.8 to 2.1)	0.012
Total body FM%	39.6 (36.7 to 42.6)	39.4 (36.7 to 42.1)	39.0 (36.4 to 41.6)	38.9 (36.2 to 41.6)	0.37
Total body LBM (SDS)	−2.1 (−2.4 to −1.8)	− 2.0 (− 2.3 to − 1.7)	−1.9 (− 2.3 to − 1.6)	−1.9 (− 2.3 to − 1.6)	0.15
Total body LBM (kg)	39.8 (37.4 to 42.2)	40.7 (38.4 to 43.0)	41.2 (38.8 to 43.5)	40.7 (38.4 to 43.0)	0.17
BMI SDS	0.9 (0.5 to 1.3)	0.9 (0.5 to 1.3)	0.9 (0.5 to 1.3)	0.9 (0.5 to 1.2)	0.60
BMI PWS SDS	−1.6 (−1.9 to − 1.2)	−1.6 (− 2.0 to − 1.2)	−1.7 (− 2.0 to − 1.3)	−1.7 (− 2.1 to − 1.3)	0.08
IGF-I SDS	1.3 (1.0 to 1.6)	1.5 (1.1 to 1.9)	1.2 (0.8 to 1.6)	1.2 (0.8 to 1.6)	0.49

Data are expressed as estimated means (95% CI). FM SDS, FM% SDS and LBM SDS were calculated according to age- and sex-matched Dutch references [25]

*P-value of the change during 3 years of GH treatment

#### Correlations in the total group

Neither FM% SDS nor LBM SDS correlated with IGF-I SDS at any time point during the 3 year study. There were no significant differences in total body FM% SDS or total body LBM SDS between the different genetic subtypes at the various time points.

### Safety

Three years of continuous GH treatment was very well tolerated (Table 3). No (S)AEs considered to be GH-related were observed. Estimated mean (95% CI) fasting glucose and insulin remained stable during the 3-year study, glucose being 4.6 (4.4 to 4.8) mmol/l at start and 4.6 (4.5 to 4.7) mmol/l after 3 years of GH, *p* = 0.93, and insulin being 59.5 (42.2 to 81.5) pmol/l at start and 55.0 (42.4 to 69.2) pmol/l after 3 years, *p* = 0.54. Systolic and diastolic blood pressure did not change significantly during 3 years of GH. None of the patients were treated with antihypertensive medication and none of the patients developed type 2 diabetes mellitus during the study.

**Table 3:** Safety parameters in total group during the 3-yar study

	At start	After 1 year	After 2 years	After 3 years	*P*-value*
Fasting glucose (mmol/l)	4.6 (4.4 to 4.8)	4.7 (4.5 to 4.9)	4.8 (4.6 to 4.9)	4.6 (4.5 to 4.7)	0.93
Fasting insulin (pmol/l)	59.5 (45.2 to 81.5)	67.6 (55.0 to 81.5)	64.5 (51.6 to 78.7)	55.0 (42.4 to 69.2)	0.54
Systolic BP (mmHg)*	120.2 (116.5 to 123.9)	123.9 (120.6 to 127.1)	122.7 (118.6 to 126.8)	121.4 (116.4 to 126.3)	0.68
Diastolic BP (mmHg)*	73.1 (70.5 to 75.7)	73.0 (71.1 to 75.0)	74.3 (72.0 to 76.5)	73.6 (70.9 to 76.3)	0.78

Data are expressed as estimated means (95% CI). *BP* Blood pressure. *corrected for sex and height

*P-value of the change during 3 years of GH treatment

### Subanalyses of changes in the continuation group and restart group

#### Changes in the year prior to the 3-year study in patients who continued GH treatment

The continuation group consisted of 17 patients (10 males, 7 females). In this group, the median (IQR) age at GH dose lowering after AH was 17.7 (17.1 to 18.4) years for males and 15.9 (14.8 to 17.4) years for females. Adult height (AH) was − 0.4 (− 1.7 to − 0.2) SDS and BMI 22.0 (19.7 to 25.4) kg/m^2^, being 0.6 (− 0.1 to 1.5) SDS. Six (35.3%) patients had a deletion, nine (52.9%) an mUPD and two (11.8%) an ICD.

**Fig. 2:**
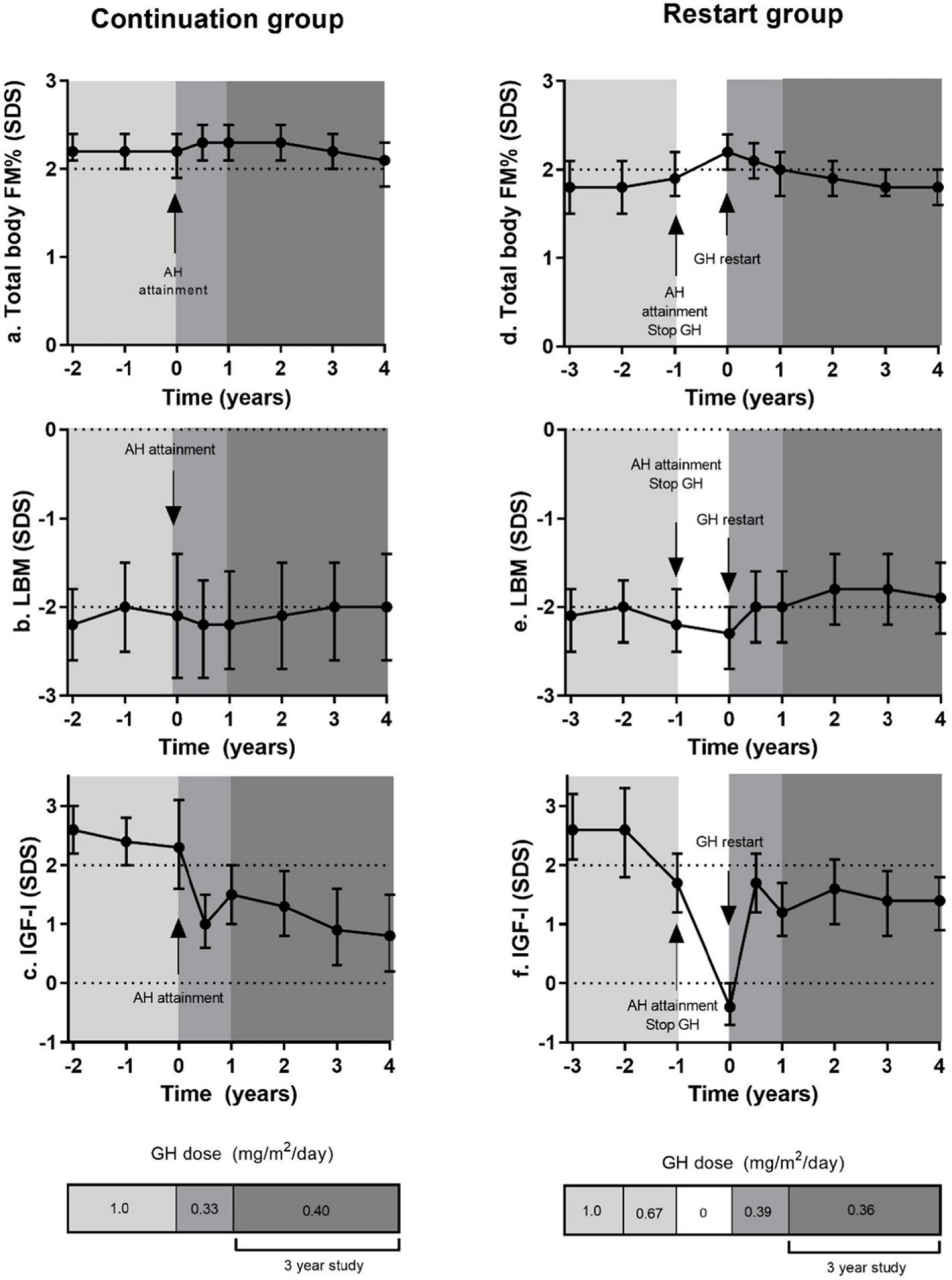
**a**-**f**.Changes in body composition after AH attainment and during the last years before AH attainment in the group of 17 young adults with PWS, who continued GH after AH (**a**-**c**) and the 26 young adults with PWS, who restarted GH treatment after AH attainment (**d**-**f**). Presented as Estimated Marginal Means with 95% CI. GH dose expressed as median dose in mg/m^2^/day

Figure 2a-c shows the changes in median GH dose and body composition and IGF-I SDS, in estimated marginal means, during GH treatment in the 17 patients who continued GH after AH attainment. All subjects were treated with GH during childhood, with a median (IQR) GH dose at AH attainment of 1.0 (0.67 to 1.0) mg/m^2^/day and estimated mean (95% CI) IGF-I SDS was 2.4 (1.6 to 3.2) SDS. After AH attainment, the GH dose was reduced to 0.33 mg/m^2^/day and in case of IGF-I SDS levels below or above our target of 1 to 2 SDS, the GH dose was adjusted. After 1 year, estimated mean (95% CI) IGF-I SDS was 1.5 (1.0 to 2.0) SDS, which was significantly lower than the 2.4 (1.6 to 3.2) SDS at AH attainment, *p* = 0.014. Following dose lowering, estimated mean (95% CI) total body FM% SDS increased significantly from 2.2 (1.9 to 2.4) SDS at AH attainment to 2.3 (2.1 to 2.5) SDS at 6 months after dose lowering, *p* = 0.009. The next 6 months of treatment FM% SDS remained stable. Estimated mean (95% CI) total body LBM SDS decreased following dose lowering from − 2.2 (− 2.8 to − 1.6) SDS to − 2.3(− 2.8 to − 1.8) SDS after 6 months, but this decrease was not significant (*p* = 0.26). The next 6 months of treatment LBM SDS remained stable.

#### Changes in the year prior to the 3-year study in patients who restarted GH treatment

The restart group consisted of 26 patients (8 males, 18 females), of which 21 had participated in the Transition study [14]. Median (IQR) duration of cessation of GH treatment was 1 (1 to 1) year. In this group, median (IQR) age at GH restart was 19.8 (19.2 to 21.1) years for males and 19.1 (16.2 to 20.1) years for females, and adult height was − 1.1 (− 1.8 to − 0.7) SDS and BMI 24.8 (20.3 to 28.9) kg/m^2^, being 1.2 (− 0.5 to 2.2) SDS. Twelve (46.1%) had a deletion, 11 (42.3%) an mUPD, two (7.6%) an ICD and one (3.8%) a translocation.

Figure 2d-f shows the changes in median GH dose and estimated marginal mean body composition and IGF-I SDS in the 26 patients who restarted GH. At restart, after cessation of GH treatment for a median duration of 1 year, estimated mean (95% CI) IGF-I SDS was − 0.4 (− 0.7 to 0.0) SDS. GH treatment was restarted in a median (IQR) dose of 0.33 (0.33 to 0.35) mg/m^2^/day. After 1 year, IGF-I SDS was 1.2 (0.8 to 1.7) SDS, which was significantly higher than at restart, *p* < 0.001. Estimated mean (95% CI) FM% SDS after 1 year without GH was 2.2 (2.0 to 2.4) SDS, during the first 6 months of GH, there was a significant decrease in FM% SDS to 2.1 (1.9 to 2.3) SDS, *p* = 0.03, which decreased further during the next 6 months to 2.0 (1.7 to 2.2) SDS, *p* = 0.02. LBM SDS at restart of GH was − 2.3 (− 2.7 to − 2.0) SDS. During the first 6 months of GH there was a significant increase of LBM SDS to − 2.0 (− 2.5 to − 1.6) SDS, *p* = 0.008, which remained stable during the next 6 months of GH (− 2.0 (− 2.4 to − 1.6) SDS).

## Discussion

To our knowledge, this is the first prospective study to describe the effects of 3 years of continuous GH treatment with a stable GH dose in young adults with PWS who were treated with GH for several years during childhood. Our findings demonstrate that GH treatment in young adults with PWS has sustained positive effects on body composition, maintaining the improved FM% SDS and LBM SDS attained during childhood. We did not find any GH-related adverse events.

Long-term GH treatment during childhood has well-known beneficial effects on body composition [5]. Present study describes the beneficial effects of 3 years of GH treatment in young adults with PWS who were treated with GH for several years during childhood. We found that longer-term GH treatment keeps FM% SDS and LBM SDS stable. These results are in line with our previous study regarding the effects of GH treatment in children with PWS, in which longer-term GH treatment had also sustained positive effects and led to a stable body composition [5].

We did not find any changes in BMI SDS during 3 years of GH treatment, which is in agreement with the 1 year studies [16–20]. We emphasize that measurement of body composition in patients with PWS is much more informative than BMI, since BMI was within normal ranges for most patients throughout our study, while body composition was abnormal. BMI is, therefore, not an appropriate parameter in PWS.

Blood pressure did not significantly change during the 3-year study and we did not find any GH-related adverse events. Fasting glucose and insulin levels remained stable. This suggests that the GH dose used in our study is also safe on the longer-term term. However, very long-term data are required to find which GH dose is optimal for a most favorable effect on body composition with the lowest possible risk for adverse events.

In patients who restarted GH, after discontinuation for a median duration of 1 year, we found a decrease of FM% SDS and an increase in LBM SDS during the first year of GH treatment compared to measurements after 1 year of GH discontinuation. Therefore, restart of GH is able to recover the positive effects on body composition after a deterioration during 1 year of GH discontinuation.

Other studies investigating 1 year of GH treatment also showed positive effects on body composition [15–19]. A study by Butler et al., in 11 GH-naïve adults with PWS, found an improvement of body composition during 1 year of GH treatment. In that study, 1 year of GH treatment was followed by 1 year observational period without GH treatment. After that year, the young adults had regained more fat mass than they lost during 1 year GH treatment [17]. Our previous randomized, double-blind, placebo-controlled, cross-over trial, also showed a deterioration of FM during 1 year of placebo, while 1 year of GH maintained the improved body composition attained during childhood [14]. Our present study demonstrates that GH treatment for longer time maintains the improved body composition in young adults with PWS.

Only one study by Sode-Carlsen et al. investigated the effects of 2 years of GH treatment in 39 young adults with PWS, but GH treatment during childhood was not recorded [20]. They report an increase in LBM of 2.8 kg after 2 years of GH, which is in line with our study. Unfortunately, the change in FM% was not reported in the study, but they describe a decrease in total FM of 3.0 kg after 2 years of GH. As in our study, they reported no serious or unexpected adverse events [20].

In the patients who continued GH, we found an increase in FM% SDS during the first 6 months after AH attainment in the group who immediately continued GH after AH attainment. An explanation for this increase could be that, after AH attainment, the GH dose was lowered from 1 mg/m^2^/day to 0.33 mg/m^2^/day. This suggests that GH stop, restart or dose lowering is followed by a change in body composition.

Since the young adults in our study had attained adult height, we did not want to continue the childhood dose of 1.0 mg/m^2^/day. We, therefore, chose a starting dose of 0.33 mg/m^2^/day after AH attainment and we titrated the GH dose to IGF-I levels between 1 and 2 SDS. During the 3-year study the median dose was 0.38 mg/m^2^/day, which corresponded to a median daily dose of 0.70 mg, which is in line with other studies in young adults [15, 16, 18–20]. In order to investigate the effects of a stable dosage of GH in the total group, we decided to only use measurements after 1 year of GH in a dose of 0.33 mg/m^2^/day as starting point for the 3-year study. At that moment, IGF-I SDS was 1.3 (1.0 to 1.6) SDS at a median daily GH dose of 0.70 mg. GH dose and IGF-I SDS values remained stable during the 3-year study, which allowed us to investigate the effects of 3 years of GH treatment with a stable GH dose. The Growth Hormone Research Society Workshop published a consensus guideline for GH treatment in PWS and suggested that a serum IGF-I level of 0 to 2 SDS would be optimal [29]. During our 3-year study, IGF-I levels remained within these ranges.

Our study did not include a control group of PWS patients who did not receive GH treatment. Since our previous GH-placebo study showed a deterioration of body composition during 1 year of placebo [14], it was considered unethical to withhold GH treatment for a longer period in these young adults. Studies in adults not treated with GH reported FM percentages of at least 50% [17, 18, 30], while in our present study the estimated mean FM% at the end of the 3-year study was 38.9%.

## Conclusion

In conclusion, our 3-year study in 43 young adults with PWS who were treated with GH during childhood shows that GH treatment maintains the improved body composition attained during childhood. Furthermore, it shows that restart of GH after 1 year of discontinuation is able to restore the deteriorated body composition with a decrease in FM% SDS and LBM SDS during the first year and sustained effects thereafter. No major side effects or safety concerns were seen. Based on our findings, we conclude that adult patients with PWS benefit from GH treatment.

## Data Availability

The datasets generated and/or analysed during the current study are not publicly available due to privacy and ethical restrictions but are available from the corresponding author on reasonable request.
